# Clinical and screening utility of the Burnout Assessment Tool: A comparative evaluation of BAT23, BAT12 and BAT4 in Sweden

**DOI:** 10.5271/sjweh.4286

**Published:** 2026-05-01

**Authors:** Emina Hadžibajramović, Isabelle Dahlqvist, Ingibjörg H Jonsdottir, Hans De Witte

**Affiliations:** 1 Institute of Stress Medicine, Region Västra Götaland, Gothenburg, Sweden.; 2 School of Public Health and Community Medicine, Institute of Medicine, Sahlgrenska Academy at the University of Gothenburg, Gothenburg, Sweden.; 3 O2L, Research group Work, Organizational and Personnel Psychology (WOPP), FPPW, KU Leuven, Belgium.; 4 Optentia Research Unit, North-West University, South Africa.

**Keywords:** burnout diagnosis, clinical burnout, exhaustion disorder, clinical cut-off, screening, ROC analysis

## Abstract

**Objectives:**

Clinical burnout may have serious personal and societal impacts, such as reduced productivity, prolonged sick leave and long rehabilitation. Regular screening using clinically relevant cut-offs to identify individuals at risk is essential for effective prevention. This study aimed to: (i) establish Swedish clinical cut-off scores for the Burnout Assessment Tool (BAT) versions (ie., BAT23, BAT12, BAT4); (ii) assess the BAT4’s ability to classify burnout severity; and (iii) calculate burnout complaints in the Swedish workforce.

**Methods:**

A national representative sample of the Swedish working population (N=1603) and a burned-out group (N=159) diagnosed with exhaustion disorder (ED) were surveyed using BAT23, BAT12, and BAT4. Receiver operating characteristic (ROC) analyses determined diagnostic accuracy and cut-offs for mild (orange) and severe (red) burnout complaints. A sensitivity analysis using clinician-confirmed ED patients (N=25) validated findings.

**Results:**

BAT23 showed the highest accuracy in differentiating between mild and severe complaints. Among the subscales, exhaustion performed best. BAT12 showed good accuracy at the orange and slightly reduced sensitivity at the red cut-off. BAT4, while showing excellent sensitivity (0.93) at the orange cut-off, had poor sensitivity (0.47) at the red cut-off, limiting its clinical utility. Prevalence estimates using Swedish cut-offs showed approximately 13% of the workforce had severe burnout complaints (BAT23 and BAT12).

**Conclusions:**

BAT23 is recommended for comprehensive assessments; BAT12 is useful for workplace screening where a shorter questionnaire is required and BAT4 for broad organizational screening using only the orange cut-off. Findings support the structured application of BAT across varying settings and a deeper understanding of the BAT4.

Burnout was introduced in the 1970s (1) and is related to huge costs at individual, organizational and societal levels (2). These costs are particularly evident in welfare states, where social funds cover the costs of sickness absence and work incapacitation. Burnout is recognized in the 11^th^ Revision of the International Classification of Diseases (ICD-11) as an occupational phenomenon, defined as a syndrome resulting from chronic workplace stress that has not been successfully managed (3). In the present study, we conceptualize burnout using the Burnout Assessment Tool (BAT), which defines burnout as a work-related state characterized by extreme tiredness, reduced ability to regulate cognitive and emotional processes, and mental distancing, accompanied by secondary psychological and psychosomatic complaints (4).

Burnout undermines an individual’s ability to cope with stress, perform effectively, and maintain overall well-being, thereby negatively affecting mental health, as defined by the World Health Organization (WHO). Importantly, mental health is more than just the absence of mental disorders; it should be understood as a complex continuum ranging from mental well-being to mental health problems and mental disorders (5).

The term burnout is often used to describe both mental health problems and a mental disorder, thus distinguishing between *burnout complaints* and the clinical condition often called *clinical burnout* is essential when discussing these conditions (6, 7). Burnout complaints refer to mental health problems, ranging from mild to severe symptoms. Mild burnout complaints may not impair daily functioning or work performance, and one can often recover naturally over time. However, if left unaddressed, burnout complaints may progress to severe symptoms and gradually develop into a mental disorder called *clinical burnout,* consistent with clinical perspectives and professional guidelines that have been developed nationally (eg, the Dutch work-related mental health guidelines) (7, 8) with serious consequences, including reduced productivity, long-term sickness absence and, in many cases, a need for long rehabilitation (9). For that reason, effective preventive measures, including regular screening of burnout complaints to detect individuals at risk, are recommended.

Burnout complaints are usually measured using self-report questionnaires. Over the past five decades, numerous definitions and tools have been developed, yet many lack comprehensive evidence of psychometric validity (10–13). For valid and reliable assessments, questionnaires must be built on a strong theoretical framework using a well-defined construct. The Burnout Assessment Tool (BAT) meets these criteria as it is grounded in an updated definition in which burnout is a work-related state of mental exhaustion characterized by extreme tiredness, cognitive and emotional impairment, and mental distancing (4).

Three versions of the BAT have been developed: a long (BAT23), a short (BAT12), and an ultra-short (BAT4) version, allowing for flexibility in different contexts (4, 14, 15). A recent review by Schaufeli & De Witte (16), summarizing data from over 40 studies across various countries, confirmed strong internal consistency for BAT23 and BAT12.

The construct validity of the BAT4 has been assessed using representative samples from eight countries (15). More recently, the BAT4 was also evaluated in a representative sample of the Swedish workforce, alongside the BAT23 and the BAT12, with findings confirming that the items and factor structure align well with the theoretical burnout construct, demonstrating good internal construct validity for all three versions for use in the Swedish context (manuscript under review).

Beyond construct validity, the practical usability of burnout questionnaires depends on the availability of normative data and clinically validated cut-off scores.

Normative data allows for benchmarking across groups, such as comparing workplaces or organizations, with the national average. Clinically validated cut-off scores are essential for identifying individuals at risk of clinical burnout through preventive screening. Without clearly defined cut-offs interpretation of scores becomes less precise, limiting the tool’s utility in identifying individuals at risk or in need of intervention.

Clinical cut-offs for the BAT23 and the BAT12 have been developed using representative samples and clinical burnout patient data from three European countries: The Netherlands, Belgium (Flanders), and Finland (17). Based on a traffic-light model, three levels were proposed indicating the degree of burnout complaints (and the risk of clinical burnout): green – no complaints (low risk), orange – mild complaints (at risk) and red – severe complaints (at a very high risk).

While pooled cut-off scores from these three countries are useful for an initial assessment, country-specific cut-offs are preferable for greater accuracy, as levels of reported burnout complaints and their interpretation can vary across countries due to cultural, healthcare, and societal differences. Norms for what constitutes “mild” or “severe” complaints may differ, as may the threshold at which symptoms are clinically significant. To the best of our knowledge, the potential of the BAT4 to differentiate between varying levels of burnout complaints has not yet been investigated.

The aim of the present study was to establish clinical cut-off scores for the BAT23 and BAT12 in a Swedish context. An additional aim was to investigate the feasibility of establishing clinical cut-off scores for the BAT4. A third aim was to calculate the burnout complaints levels in the Swedish working population.

## Method

### Study population and procedure

The survey data in the present study was part of a larger research project with the overall aim of increasing knowledge concerning patients seeking care for stress-related exhaustion. Data were collected using an internet-based survey delivered by a commercial sampling company in Sweden to: (i) a randomly selected national representative sample of the working population in Sweden and (ii) self-recruited participants that had sought care for stress-related exhaustion/symptoms. The target population for both surveys comprised adults aged 18–74 years.

For the purpose of the study, all participants were asked the following questions: “*Have you sought care for stress-related exhaustion/symptoms during the past five years*?” (*Yes/No*); “*Did you receive a diagnosis when you sought care*? (*Yes, exhaustion disorder/Yes, another/No diagnosis/Do not know*); “*Which option applies to you currently*” (*I am currently experiencing ongoing stress-related exhaustion/ I no longer have stress-related exhaustion but still have some symptoms or difficulties/ I no longer have stress-related exhaustion and feel fully recovered*).

The Swedish National Board of Health and Welfare established exhaustion disorder (ED) as a criteria-based diagnosis (18) similar to clinical burnout as defined by the Royal Dutch Medical Association in the guidelines for work-related mental health issues (7, 19). The condition is characterized by physical and mental symptoms of exhaustion, markedly reduced mental energy, memory impairment, sleep disturbance, emotional instability, and intolerance to stress. Most patients with ED report high levels of burnout complaints (20).

The participants answered the work-related version of the BAT if they were currently employed (working full- or part-time). Those currently not working (eg, unemployed, on full-time parental leave or similar) were asked to answer the general non-work-related version of the BAT that consists of context-free items that do not refer to a work-setting.

### National representative sample of the Swedish workforce

Data were collected during May–June 2023. The study questionnaire was distributed to a random sample of individuals drawn from a survey company’s panel of 100 000 randomly recruited active panel members. The panel reflects the Swedish population regarding age, gender and region. The company follows the ESOMAR guidelines for web panel management to ensure transparency and quality (*esomar.org/uploads/attachments/clftgzsxu06o27g3vgdlhdpwd-37-questions-updated-version.pdf)*.

Quota sampling for age and gender based on official Statistics Sweden data (Labor Force Surveys) was applied to ensure the representativeness of the Swedish workforce. Stratified random samples were selected within each age and gender group, and data collection for each group was closed once the predetermined number of participants was reached.

In total, responses from N=2014 participants were collected. Among those, N=1603 had complete answers on all BAT work-related items and were used for calculation of prevalences (aim 3).

For the first two aims, eligible to be included as the supposedly healthy control group (the so-called 'healthy employee sample') consisted of participants who had not sought care for stress-related symptoms during the past five years (N=1446). This designation did not imply complete absence of symptoms but served as a proxy for non-clinical status for receiver operating characteristic (ROC) analyses. Individuals in this group still may have reported burnout complaints. Ineligible participants were those who: (i) sought care for stress-related symptoms during the previous five years (N=545); (ii) did not answer the question about seeking care (N=13); (iii) did not answer the question about current employment (N=10). The analyses included 1162 eligible participants who had complete answers on all BAT work-related items (excluded: 249 respondents to the BAT-general version who were not employed either full- or part-time and 35 who were missing ≥1 items on the BAT work-related version). The age and gender distributions in the healthy employee sample were comparable to those of the overall Swedish workforce, with deviations across age and gender strata of 0.4–3.5 percentage points, which were considered acceptable. Mean age in the healthy employee sample was 43.7 [standard deviation (SD) 12.45] years and the percentage women/men was 40/60.

### Burned-out group

The group of participants that we called the 'burned-out group' consisted of self-recruited participants who had sought care for stress-related symptoms within the previous five years. Data was collected from May to October 2023. Self-recruited participants (N=745) were approached via a snowball method through (i) advertisements in social media and through nationally distributed, strategically placed posters and flyers in clinical waiting rooms (sample 1: N=684); and (ii) care-giving personnel distributing flyers with study information to ED-diagnosed patients (sample 2: N=61).

In the present study, the inclusion criteria for the burned-out group were that a participant (i) was diagnosed with ED, (ii) was currently experiencing stress-related exhaustion/symptoms and (iii) had complete answers on the BAT work-related items. In total N=159 participants fulfilled these criteria, mean age 48.3, SD=9.4 years (sample 1: N=134 and sample 2: N=25).

### Ethical approval

Prior to completing the questionnaire, all participants were informed about the purpose of the study, that the data would be collected anonymously and that participation was voluntary. They all gave written consent to participate. The study was conducted in compliance with the Declaration of Helsinki and approved by the Swedish National Authority for Ethical Approval in March 2023 (approval number 2022-07190-01).

### Burnout complaints

Burnout complaints are measured with the work-related BAT. The BAT23 consists of 23 items divided into four subscales: exhaustion (EX, 8 items), cognitive impairment (CI, 5 items), emotional impairment (EI, 5 items), and mental distancing (MD, 5 items) (4). The BAT12 consists of three items for each subscale (14) and the BAT4 consists of four items, one for each subscale (15). All items are expressed as statements responded on a frequency-based scale ranging from “never” to “always”. For all three versions, a mean score of all items is calculated to indicate a total burnout level (range 1–5, higher scores indicated higher burnout levels). For the BAT23, mean scores for each subscale were also calculated.

### Data analysis

Descriptive statistics for all study variables are presented in terms of median and interquartile range (Q1 and Q3), observed minimum and maximum values. Given the ordinal data, comparisons between different groups were tested by the Mann-Whitney U test.

ROC-curve analyses were conducted to determine optimal cut-off values on the BAT for differentiating between the burned-out group and the healthy employees. ROC curves were generated for the total scores of the BAT23, BAT12, and BAT4, and each BAT23 subscale. This method evaluates the diagnostic accuracy by plotting sensitivity (true positive rate) against 1-specificity (false positive rate) across all possible thresholds (21).

The diagnostic accuracy of the BAT was assessed using the area under the curve (AUC), sensitivity and specificity. The AUC range is 0–1, with higher values indicating greater discriminative ability between true positives (burned-out individuals) and true negatives (healthy employees). In general, an AUC of 0.5 suggests no better than chance discrimination, while 0.7 ≤AUC<0.8 is considered acceptable, 0.8≤AUC<0.9 is excellent and ≥0.9 is outstanding (22).

Swedish cut-off values were established using the traffic light model proposed by Schaufeli et al (reference?), categorizing individuals according to the degree of burnout complaints (and the risk of ED): green – no complaints (low risk for ED), orange – mild complaints (at risk for ED) and red – severe complaints (at a very high risk for ED). A specificity threshold of >0.90 was set as a criterion for the red category to ensure the BAT’s clinical suitability. High specificity is crucial for confirmatory diagnostics, as it prioritizes accurately ruling out individuals who are not affected, minimizing false positives. In other words, misclassification healthy individuals as burnout cases for clinical evaluation should be low: <10% in this case. An acceptable level for sensitivity was set at ≥0.70. The orange cut-off was determined using the Youden index (23). This index is calculated by deducting 1 from the sum of sensitivity and specificity and ranges 0–1 (the higher the better discriminative ability of the instrument). The threshold for the orange cut-off was decided at the point where the Youden index reached its maximum, which provides an optimal balance for screening purposes in organizations, where besides specificity also sensitivity is crucial for accurately detecting potential mild complaints and enabling early intervention (acceptable level ≥0.70).

*Sensitivity analysis.* Although all participants in the burned-out group (N=159) reported having been diagnosed with ED by a healthcare professional after seeking care for stress-related exhaustion and were currently experiencing stress-related exhaustion symptoms, a sensitivity analysis was conducted to assess the robustness of the results. In this analysis, a subset of participants (N=25) whose ED diagnosis was additionally confirmed by the healthcare professional who invited them to participate in the survey was compared with both the healthy employee sample and the remaining burned-out participants (N=134).

Any deviations between the cut-off scores obtained in the main analysis and the sensitivity analysis were reported. Additional analyses were done to compare the pooled cut-offs (17) with Swedish cut-offs for the BAT23 and BAT12 (pooled cut-offs are not available for the BAT4). Comparisons were made by cross-tabulating the participants into the three risk groups (green, orange, red) and calculating the percentage agreement (ie, percentage of the same classification by both cut-off scores) as well as the percentage disagreement. Differences in classifications were tested using the marginal homogeneity test (MH) test (Z=standardized MH statistic and P-value were presented, significance level <0.05).

## Results

Descriptive statistics for all study variables across the two study groups and the subgroup included in the sensitivity analysis are presented in table 1. Across all variables, the healthy employee sample reported significantly lower burnout levels compared to both the burned-out group and the subgroup of patients with confirmed clinical diagnosis (P<0.001 for all comparisons). Within the burned-out group, when divided into those with self-reported ED (N=134) and those with confirmed ED (N=25), no significant differences were observed between the groups, except for the subscale CI (P=0.031) (see supplementary material, https://www.sjweh.fi/article/4286, appendix 1).

**Table 1:** Descriptive statistics for the healthy employee sample, burned-out sample and subgroup used in the sensitivity analysis. [BAT=Burnout Assessment Tool]

		Healthy ^2^ (N=1162)			Burned-out ^3^ (N=159)			SUBGROUP Patients ^4^ (N=25)	
		Median (Q1–Q3)	Min–Max		Median (Q1–Q3)	Min–Max		Median (Q1–Q3)	Min–Max
BAT23 ^1^									
	Total score	1.87 (1.57–2.30)	1.00–4.57		3.09 (2.70–3.48)	1.96–4.65		2.96 (2.70–3.35	1.96–4.00
	Exhaustion	2.13 (1.75–2.75)	1.00–4.75		3.88 (3.38–4.25)	2.00–5.00		3.75 (3.31–3.94)	2.63–5.00
	Mental distance	1.80 (1.20–2.40)	1.00–5.00		2.60 (2.00–3.00)	1.00–4.40		2.60 (2.00–2.80)	1.00–3.60
	Cognitive impairment	2.00 (1.55–2.40)	1.00–5.00		3.20 (2.80–3.80)	1.00–5.00		3.20 (2.70–3.50)	2.20–3.80
	Emotional impairment	1.40 (1.00–2.00)	1.00–4.20		2.40 (1.80–3.20)	1.00–4.80		2.40 (1.80–3.00)	1.00–4.80
BAT12 ^1^									
	Total score	1.83 (1.50–2.25)	1.00–4.50		2.92 (2.50–3.33)	1.58–4.83		2.75 (2.50–3.13)	1.58–4.00
BAT4 ^1^									
	Total score	2.00 (1.50–2.50)	1.00–4.75		3.00 (2.75–3.50)	1.00–4.75		3.00 (2.75–3.63)	1.75–4.25

^1^ Long (BAT23) short (BAT12) and ultra-short (BAT4) version. ^2^ Reported that they had not sought care for stress-related symptoms during the previous 5 years. ^3^ Reported that they had sought care for stress-related symptoms, had been diagnosed with exhaustion disorder diagnosis and had ongoing exhaustion. ^4^ Diagnosis exhaustion disorder confirmed by a healthcare professional).

**Figure 1:**
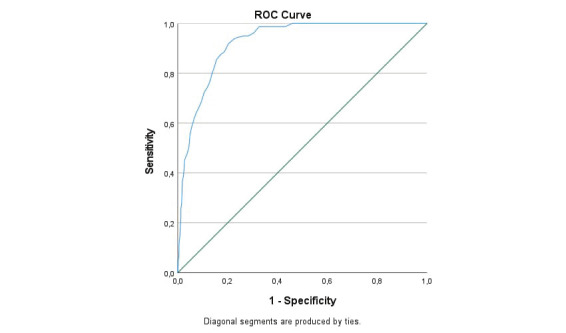
ROC-curve for the long version of the Burnout Assessment Tool (BAT23) in healthy employee groups (N=1162) and burned-out group (N=159).

The AUC for the three versions of the BAT is presented in table 2. With the exception of the MD subscale (which demonstrated acceptable accuracy), the AUC point estimates for all other variables were either outstanding (≥0.90) or excellent (≥0.80) in both the main and the sensitivity analyses. The lower boundaries for 95% confidence intervals were ≥0.80 for all measures expect for emotional impairment. A visualization of the AUC for BAT23 is provided in figure 1.

**Table 2:** Area under the curve (AUC) and 95% confidence interval (CI) for the Burnout Assessment Tool (BAT).

		Main analysis ^2^		Sensitivity analysis ^3^
		AUC (95% CI)		AUC (95% CI)
BAT23 ^1^				
	Total score	0.92 (0.91–0.94)		0.91 (0.88–0.95)
	Exhaustion	0.94 (0.93–0.96)		0.94 (0.91–0.97)
	Mental distance	0.73 (0.69–0.76)		0.72 (0.64–0.80)
	Cognitive impairment	0.91 (0.89–0.93)		0.89 (0.85–0.93)
	Emotional impairment	0.80 (0.76–0.84)		0.80 (0.72–0.89)
BAT12 ^1^				
	Total score	0.90 (0.88–0.92)		0.89 (0.83–0.94)
BAT4 ^1^				
	Total score	0.89 (0.87–0.92)		0.89 (0.84–0.95)

^1^ Long (BAT23) short (BAT12) and ultra-short (BAT4) version. ^2^ Included burned-out participants (N=159) reported that they had sought care for stress-related symptoms and health employees reported that they had not sought care for stress-related symptoms during the previous 5 years (N=1162). ^3^ Included patients diagnosis exhaustion disorder confirmed by a healthcare professional N=25) and healthy employees reported that they had not sought care for stress-related symptoms during the previous 5 years (N=1162).

The Swedish cut-off values and corresponding sensitivity and specificity for all three versions of the BAT are presented in table 3. The BAT23 demonstrated the best balance between sensitivity and specificity. The orange cut-off showed high sensitivity (0.92) and good specificity (0.80). The red cut-off was predefined to have high specificity (0.91), while its sensitivity (0.69) fell just below the acceptable threshold of ≥0.70.

**Table 3:** Swedish cut-off values for the long (BAT23), short (BAT12) and ultra-short (BAT4) versions of the Burnout Assessment Tool and its subscales. **Bold denotes <0.70**. Orange=mild complaints (at risk for clinical burnout), red=severe complaints (very high risk for clinical burnout). [SENS=sensitivity, SPEC=specificity].

Cut-off		Value	SENS	SPEC
**BAT23** ^1^				
Total				
	Orange	2.41	0.92	0.80
	Red	2.80	**0.69**	0.91
Exhaustion (EX)				
	Orange	2.94	0.92	0.84
	Red	3.31	0.79	0.91
Mental distance (MD)				
	Orange	1.70	0.86	**0.50**
	Red	3.10	**0.23**	0.92
Cognitive impairment (CI)				
	Orange	2.50	0.91	0.79
	Red	3.10	**0.62**	0.94
Emotional impairment (EI)				
	Orange	1.90	0.74	0.75
	Red	2.50	**0.46**	0.91
**BAT12** ^1^				
Total				
	Orange	2.21	0.91	0.74
	Red	2.71	**0.60**	0.91
**BAT4** ^1^				
	Orange	2.38	0.93	0.72
	Red	3.13	**0.47**	0.93

^1^ Long (BAT23) short (BAT12) and ultra-short (BAT4) version.

Among the subscales, EX performed best, demonstrating high specificity and sensitivity for both cut-offs. The subscale MD did not perform well, with the orange cut-off showing low specificity (0.50) and the red cut-off exhibiting notably low sensitivity (0.23). Subscales EI and CI showed good accuracy with the orange cut-off but had sensitivity below acceptable values for the red cut-off.

The BAT12 performed slightly below the BAT23, showing good sensitivity and specificity but with some loss of accuracy. For the red cut-off sensitivity was 0.60, while the sensitivity for the orange cut-off remained high, and had acceptable specificity (0.74).

Finally, results for the BAT4 showed that the sensitivity for the red cut-off was 0.47, indicating a greater risk of missing true burnout cases compared to the BAT23 and the BAT12, while the orange cut-off had a high sensitivity (0.93) and acceptable specificity (0.72). Thus, classification into two rather than three risk groups (ie, green vs orange) is recommended for the BAT4.

In the sensitivity analysis performed on the subsample of N=25 ED patients, the cut-off values remained the same in the main analysis for most measures (9 out of 14 variables). For the remaining measures, score differences of 0.04–0.26. Sensitivity and specificity values were comparable, except for the red cut-off for MD, where sensitivity dropped to 0.12 (see supplementary appendix 1).

Prevalences of burnout complaints in a sample of Swedish workforce are presented in table 4. The red cut-off seems stable across BAT23 and BAT12 as approximately 13% were classified as having severe complaints by both versions. However, fewer participants are classified as green and more as orange by both BAT12 and BAT4 compared to the BAT23.

**Table 4:** Percentages and counts of burnout complaints (and risk for clinical burnout) in a representative sample of Swedish workforce (N=1603); green=no complaints (low risk of clinical burnout); orange= mild complaints (at risk for clinical burnout), red=severe complaints (very high risk for clinical burnout). [BAT=Burnout Assessment Tool].

	BAT23 ^1^		BAT12 ^1^		BAT4 ^1^
	% (N)		% (N)		% (N)
Green	73.3 (1177)		68.1 (1092)		65.9 (1057)
Orange	13.2 (212)		19.3 (309)		34.1 (546)
Red	13.3 (214)		12.6 (202)		-

^1^ Long (BAT23) short (BAT12) and ultra-short (BAT4) version.

Additional analysis comparing pooled and Swedish cut-offs for the BAT23 and BAT12 showed a high level of agreement (87.6% for the BAT23 and 81.0% for the BAT12). The observed disagreement was explained by the pooled cut-offs classifying 12.4% of the participants in lower risk groups for the BAT23 (pooled-Swedish cut-offs BAT23: green-orange 6.7%, orange-red 5.7%); and 19% for the BAT12 (pooled-Swedish cut-off: green-orange 13.5%, orange-red 5.5%) (see supplementary tables S1–S2). The marginal homogeneity test indicated significant disagreement for both versions (Z=14.11 for the BAT23 and Z=17.44 for the BAT12, P-values <0.0001 for both). The results suggest that the country-specific cut-offs should be prioritized in future as the statistically significant disagreement between pooled and Swedish cut-offs indicates that the pooled values do not align fully with the Swedish data.

## Discussion

The aim of the present study was threefold. Firstly, clinical cut-off scores for the various versions of the BAT in the Swedish context were established. Secondly, we evaluated, for the first time, the utility of the ultra-short version (BAT4) in identifying individuals with no, mild and severe burnout complaints. Thirdly, levels of burnout complaints in the Swedish workforce were estimated.

Overall, the results demonstrated good accuracy of all three versions of the BAT in distinguishing between the burnout levels among healthy employees and burned-out participants. The AUC values were either outstanding or excellent for the majority of variables, confirming high discriminative ability. The BAT23 demonstrated the best overall performance, achieving an optimal balance between sensitivity and specificity for both red and orange cut-offs. The BAT12 also showed good performance, albeit with a slight reduction in precision. At the red cut-off, sensitivity decreased to 0.60, suggesting a higher risk of under-identification of possible clinical burnout cases compared to BAT23. Nonetheless, its practical advantages of shorter completion time may make it suitable for screenings where time or respondent burden is a concern. While burnout is treated as a unified construct, subscales can be used for informational purposes to provide more differentiated symptom profiles. Among the four subscales of the BAT23, exhaustion performed best while the mental distance subscale did not show satisfactory accuracy. Our results align well with the previous study by Schaufeli et al (17) which established clinically validated cut-off points for the BAT23 and BAT12 based on samples from The Netherlands, Belgium, and Finland and patients diagnosed with clinical burnout.

Of particular significance, this study is the first to evaluate the accuracy of the BAT4, a highly condensed version of the BAT, to classify individuals into no, mild and severe burnout complaints. While the orange cut-off for the BAT4 showed the highest sensitivity (0.93) of all three versions, making it potentially useful as an initial screening tool, its red cut-off sensitivity was low (0.47). Results indicate a considerable risk of false negatives when using BAT4 for the purposes of identifying plausible clinical cases, highlighting its limitations in clinical settings. Due to low sensitivity, the use of the red cut-off for the BAT4 is not recommended. Therefore, classification into three levels using BAT4 does not appear feasible; only the orange cut-off is recommended. The BAT4 can be used for broad screenings in organizations with the goal to “cast a wide net” ie, to detect individuals with any/some complaints.

Swedish cut-off values were somewhat lower compared to the other three European countries.

Our supplementary analysis highlights the importance of country-specific cut-off values, accounting for contextual and cultural factors that enhance validity within a specific population. The results were somewhat surprising given the striking similarities in the diagnostic criteria for the ED and clinical burnout (19). One possible explanation is that although the Swedish burned-out group was still experiencing symptoms, they may have been at a later stage of the clinical burnout trajectory, as the timing of diagnosis was unknown. In contrast, participants in the comparison countries completed the questionnaire shortly after clinical assessment and diagnosis being set. Given that burnout symptoms fluctuate over time (24), this may have contributed to the observed differences.

Clinical cut-off scores for the Swedish ED diagnosis have previously been established using SMBQ (25) and the Karolinska Exhaustion Scale (KEDS) (26). Both use context-free statements, making them useful in clinical settings, especially for patients away from work as they allow for tracking symptom progression over time. However, a significant limitation is that both questionnaires recently faced criticism regarding their construct validity and psychometric properties (13, 27, 28). The BAT has been shown to be psychometrically superior to the SMBQ (13).

Another limitation is that KEDS’ and SMBQ’s cut-offs identify potential clinical burnout but do not distinguish between mild and severe complaints. In modern workplaces, early detection is key to prevent mental health problems from developing into diagnosable disorders. This is particularly important given the widespread chronic stress from adverse psychosocial working conditions, highlighting the need to improve mental well-being among employees (29, 30).

Detecting early signs like mild burnout complaints, enables organizations to intervene before health and performance decline. This proactive approach supports well-being, enhances efficiency and helps prevent mental health issues from escalating.

Finally, the new cut-offs were used to estimate burnout complaints within the Swedish workforce. Results indicate that both the BAT23 and BAT12 perform similarly well in identifying individuals at very high risk for clinical burnout. The higher proportion of individuals classified as non-green (ie, mild or severe complaints) in the BAT12 and BAT4 compared to BAT23 may be explained by the lower specificity of the orange cut-off in the shorter versions, increasing the likelihood of false positives and identifying more individuals as at risk. Swedish results are comparable to those in the Netherlands (13.6%) while higher than in the Flemish (7.6%) and Finnish (6.6%) working populations (16). A previous study of Swedish physicians found 4.7% with severe burnout complaints (31). However, they used the pooled cut-off from The Netherlands, Belgium and Finland, suggesting that the prevalence might be higher with Swedish-specific cut-offs.

One limitation of the present study is that all data are self-reported, and due to respondents’ anonymity, reported diagnoses could not be clinically verified or cross-checked with medical registers. To address this, we performed a sensitivity analysis for a small group recruited by the clinicians who diagnosed them with ED, finding results similar to the main analysis. While ED participants were self-recruited, which might introduce selection bias, their age and gender aligned with previous clinical studies of ED patients (9). Although the present study did not include behavioral outcomes (eg, absenteeism), a recent study showed that severe burnout complaints (BAT-23 above the red cut-off, pooled cut-off from The Netherlands, Belgium and Finland) demonstrated predictive validity. Thus, in a one-year cohort of Swedish physicians, baseline severe burnout complaints predicted higher odds of medically certified sickness absence (OR=2.57; 95% confidence interval 1.27–5.23), even after adjustment for depressive symptoms and covariates, indicating that the BAT’s red category is meaningfully linked to work-absence risk at follow-up (32).

Finally, in addition to individual-level screening, organizational strategies are essential for sustainable prevention. The BAT can be applied not only to identify individuals at risk but also at an aggregated level to monitor burnout prevalence across departments or organizations. Aggregated BAT data can help employers detect high-risk settings, prioritize interventions, and evaluate the impact of organizational changes over time. At the same time, such analyses can identify work and organizational determinants of burnout complaints, providing valuable insight for targeted interventions. Acknowledging that prevention is not an “either/or” strategy, future research should explore integrated approaches that combine individual early detection with organizational risk profiling to address both individual and organizational contributors to burnout.

### Practical implications

The red cut-off could plausibly be used as an indication of clinical condition assisting employers and clinicians to identify individuals with a high likelihood of clinical burnout. It has a specificity >0.90, meaning that over 90% of individuals without clinically significant burnout complaints are correctly identified as not being affected, minimizing false positives. This is particularly important in clinical settings to avoid unnecessary evaluation, resource allocation and patient distress. Scores above the red cut-off suggest a strong case for clinical assessment, while those scoring below are unlikely to meet criteria for clinical burnout, thus avoiding over-diagnosis. The BAT23 can preferably been used in as a screening tool in occupational health settings to identify plausible clinical cases, the BAT12 may be acceptable when some reduction in sensitivity is tolerable, whereas the BAT4cannot be used for this purpose but may serve as a very brief check-in where the sole aim is to flag mild complaints.

The orange cut-off in the BAT is intended for screening purposes, particularly in organizational or occupational health settings, where early identification of risk is important. This threshold was established using the Youden Index, a method identifying the point that maximized the BAT’s overall discriminative ability, though it does not necessarily imply an equal balance between correctly identifying true positives and true negatives. Sensitivity at this level is high, ensuring that most individuals with emerging signs of symptoms are detected, even if this comes at the cost of some false positives. In practice, the orange cut-off serves as a preventive marker, flagging individuals who may not yet meet clinical criteria for burnout but who are at increased risk and are already experiencing mild symptoms. The orange cut-off can be applied across all three BAT versions.

While BAT23 is preferred for comprehensive assessment and screening where high accuracy is required, shorter versions may be warranted in large-scale or repeated screenings. The BAT *User Manual* notes that the full BAT takes about five minutes to complete, with the short version requiring less (33); in practice, this corresponds to roughly, based on our field experience, 4–5 minutes (BAT23), 2–3 minutes (BAT12), and <1 minute (BAT4). For organizations screening thousands of employees, these differences translate into hours of administration time.

### Concluding remarks

Results from the present study support the structured application of BAT across varying settings. BAT23 is recommended for comprehensive assessment including identification of plausible clinical cases. The BAT12 is efficient and reliable for workplace screening. The BAT4 is an exploratory tool is suitable for workplace settings or large-scale contexts using only the orange cut-off. Our evaluation of BAT4 represents a valuable contribution to the field, offering a new, ultra-brief measure whose strengths and limitations are now better understood.

## Supplementary material

Supplementary material

## References

[1] Burned-out MC. Hum Behav 1976;5:16–22.

[2] Demerouti E. Burnout: a comprehensive review. Z Arbeitswiss 2024;78(4):492–504. 10.1007/s41449-024-00452-3

[3] WHO. International Classification of Diseases, Eleventh Revision (ICD-11) 2019/2021. World Health Organization (WHO) 2019.

[4] Schaufeli WB, Desart S, De Witte H. Burnout Assessment Tool (BAT)-Development, Validity, and Reliability. Int J Environ Res Public Health 2020 Dec;17(24):9495. 10.3390/ijerph17249495 33352940 PMC7766078

[5] Rugulies R, Aust B, Greiner BA, Arensman E, Kawakami N, LaMontagne AD et al. Work-related causes of mental health conditions and interventions for their improvement in workplaces. Lancet 2023 Oct;402(10410):1368–81. 10.1016/S0140-6736(23)00869-3 37838442

[6] Schaufeli W, Hakanen J, Shimazu A. Chapter 7: Burning questions in burnout research. In: De Cuyper N, Selenko E, Euwema M, Schaufeli W, editors. Job Insecurity, Precarious Employment and Burnout Job Insecurity, Precarious Employment and Burnout: Facts and Fables in Work Psychology Research: Edward Elgar Publishing; 2023. p. 127–48.

[7] Schaufeli WB, de Rijk A, Herlofson J, Åsberg M. How to Diagnose Clinical Burnout Using Dutch and Swedish Approaches? J Occup Environ Med 2025 Dec;•••: ; E-pub ahead of print. 10.1097/JOM.0000000000003644 41382323

[8] van Dam A. A clinical perspective on burnout: diagnosis, classification, and treatment of clinical burnout. Eur J Work Organ Psychol 2021;30(5):732–41. 10.1080/1359432X.2021.1948400

[9] Glise K, Wiegner L, Jonsdottir IH. Long-term follow-up of residual symptoms in patients treated for stress-related exhaustion. BMC Psychol 2020 Mar;8(1):26. 10.1186/s40359-020-0395-8 32188513 PMC7081527

[10] Guseva Canu I, Marca SC, Dell’Oro F, Balázs Á, Bergamaschi E, Besse C et al. Harmonized definition of occupational burnout: A systematic review, semantic analysis, and Delphi consensus in 29 countries. Scand J Work Environ Health 2021 Mar;47(2):95–107. 10.5271/sjweh.3935 33258478 PMC8114565

[11] Schaufeli W. The burnout enigma solved? Scand J Work Environ Health 2021 Apr;47(3):169–70. 10.5271/sjweh.3950 33604675 PMC8126437

[12] Shoman Y, Marca SC, Bianchi R, Godderis L, van der Molen HF, Guseva Canu I. Psychometric properties of burnout measures: a systematic review. Epidemiol Psychiatr Sci 2021 Jan;30:e8. 10.1017/S2045796020001134 33436137 PMC8057391

[13] Shoman Y, Hostettler R, Canu IG. Psychometric validity of the Shirom-Melamed Burnout Measure and the Burnout Assessment Tool: a systematic review. Arh Hig Rada Toksikol 2023 Dec;74(4):238–45. 10.2478/aiht-2023-74-3769 38146759 PMC10750325

[14] Hadžibajramović E, Schaufeli W, De Witte H. Shortening of the Burnout Assessment Tool (BAT)-from 23 to 12 items using content and Rasch analysis. BMC Public Health 2022 Mar;22(1):560. 10.1186/s12889-022-12946-y 35313849 PMC8939057

[15] Hadžibajramović E, Schaufeli W, De Witte H. The ultra-short version of the Burnout Assessment Tool (BAT4)-development, validation, and measurement invariance across countries, age and gender. PLoS One 2024 Feb;19(2):e0297843. 10.1371/journal.pone.0297843 38394265 PMC10889892

[16] Schaufeli W, De Witte H. Burnout Assessment Tool (BAT) - A fresh look at burnout. In: Krägeloh CU, Alyami M, Medvedev ON, editors. International Handbook of Behavioral Health Assessment. Cham: Springer International Publishing; 2023. p. 1–24.

[17] Schaufeli WB, De Witte H, Hakanen JJ, Kaltiainen J, Kok R. How to assess severe burnout? Cutoff points for the Burnout Assessment Tool (BAT) based on three European samples. Scand J Work Environ Health 2023 May;49(4):293–302. 10.5271/sjweh.4093 37042446 PMC10713992

[18] Socialstyrelsen. Utmattningssyndrom: stressrelaterad psykisk ohälsa. [English translation] Stockholm: The National Board of Health and Welfare (Socialstyrelsen); 2003. Report No.: 9172017864.

[19] Schaufeli W, De Rijk A, Herlofson J, Åsberg M. Towards diagnostic criteria for clinical burnout. Conference paper presented at European Organisation of Work and Organisational Psychology - EAWOP 2025; Prague 21-24 May 20252025.

[20] Jonsdottir IH, Hägg DA, Glise K, Ekman R. Monocyte chemotactic protein-1 (MCP-1) and growth factors called into question as markers of prolonged psychosocial stress. PLoS One 2009 Nov;4(11):e7659. 10.1371/journal.pone.0007659 19888340 PMC2766003

[21] Altman DG. Practical Statistics for Medical Research. London: Chapman & Hall/CRC; 1991.

[22] Hosmer DW, Lemeshow S. Applied logistic regression. New York; Wiley; 2000.

[23] Youden WJ. Index for rating diagnostic tests. Cancer 1950 Jan;3(1):32–5. 10.1002/1097-0142(1950)3:1<32::AID-CNCR2820030106>3.0.CO;2-3 15405679

[24] Glise K, Ahlborg G Jr, Jonsdottir IH. Course of mental symptoms in patients with stress-related exhaustion: does sex or age make a difference? BMC Psychiatry 2012 Mar;12(1):18. 10.1186/1471-244X-12-18 22409935 PMC3338076

[25] Lundgren-Nilsson Å, Jonsdottir IH, Pallant J, Ahlborg G Jr. Internal construct validity of the Shirom-Melamed Burnout Questionnaire (SMBQ). BMC Public Health 2012 Jan;12:1. 10.1186/1471-2458-12-1 22214479 PMC3307433

[26] Besèr A, Sorjonen K, Wahlberg K, Peterson U, Nygren A, Asberg M. Construction and evaluation of a self rating scale for stress-induced exhaustion disorder, the Karolinska Exhaustion Disorder Scale. Scand J Psychol 2014 Feb;55(1):72–82. 10.1111/sjop.12088 24236500 PMC4235404

[27] Lindsäter E, van de Leur JC, Rück C, Hedman-Lagerlöf E, Bianchi R. Psychometric and structural properties of the Karolinska Exhaustion Disorder Scale: a 1,072-patient study. BMC Psychiatry 2023 Sep;23(1):642. 10.1186/s12888-023-05138-4 37660017 PMC10475192

[28] Almén N, Jansson B. The reliability and factorial validity of different versions of the Shirom-Melamed Burnout Measure/Questionnaire and normative data for a general Swedish sample. Int J Stress Manag 2021;28(4):314–25. 10.1037/str0000235

[29] Silvaggi F, Miraglia M. Mental health at work: A review of interventions in organizations. E-Journal of international and comparative labour studies. 2017;6. Available from: https://ejcls.adapt.it/index.php/ejcls_adapt/article/view/443

[30] Schulte PA, Sauter SL, Pandalai SP, Tiesman HM, Chosewood LC, Cunningham TR et al. An urgent call to address work-related psychosocial hazards and improve worker well-being. Am J Ind Med 2024 Jun;67(6):499–514. 10.1002/ajim.23583 38598122 PMC11980372

[31] Hagqvist E, Ekberg K, Lidwall U, Nyberg A, Landstad BJ, Wilczek A et al. The Swedish HealthPhys Study: Study Description and Prevalence of Clinical Burnout and Major Depression among Physicians. Chronic Stress (Thousand Oaks) 2022 Apr;6:24705470221083866. 10.1177/24705470221083866 35402760 PMC8984863

[32] Brulin E, Wilczek A, Ekberg K, Lidwall U, De Beer LT, Hadzibajramovic E et al. Predictive value of burnout complaints and depressive symptoms for medically certified sickness absence among physicians in Sweden: a 1 year follow-up observational study. BMJ Open 2025 Apr;15(4):e090966. 10.1136/bmjopen-2024-090966 40216422 PMC11987104

[33] Schaufeli WB, De Witte H, Desart S. User Manual – Burnout Assessment Tool (BAT) – Version 2.0. KU Leuven, Belgium: Internal Report; 2019.

