# Supplementary material for: Clinical and screening utility of the Burnout Assessment Tool: A comparative evaluation of BAT23, BAT12 and BAT4 in Sweden
Source: Scand J Work Environ Health. 2026 Apr 30;52(3):263–71. doi: 10.5271/sjweh.4286 (PMC13182256; doi:10.5271/sjweh.4286)
Supplement: Supplementary material [file SJWEH-52-263-S001.pdf]

**Clinical and screening utility of the Burnout Assessment Tool: A comparative evaluation of BAT23, BAT12 and BAT4 in Sweden**[1]

***by Emina Hadžibajramović, PhD*** [2] ***Isabelle Dahlqvist, MSc, Ingibjörg H Jonsdottir, PhD, Hans De Witte, PhD***

1. Supplementary material
2. Correspondence to: Emina Hadžibajramović, PhD, Institute of Stress Medicine, Region Västra Götaland, Gothenburg, Sweden. [E-mail: emina.hadzibajramovic@vgregion.se]

**Table A1.** Sensitivity analysis using data from participants (n=25) for whom diagnosis exhaustion disorder was confirmed by a healthcare professional: SENS= sensitivity, SPEC=specificity, Δ= differences in cut-off values between main and sensitivity analyses; bold denotes <0.70; orange= mild complaints (at risk for clinical burnout), red= severe complaints (very high risk for clinical burnout).

| | | | Sensitivity analysis | | |
|---|---|---|---|---|---|
| | | **Cut-off** | **SENS** | **SPEC** | **Δ** |
| **BAT23**[1] | Total | Orange | 0.96 | 0.81 | 0.04 |
| | | Red | **0.64** | 0.91 | - |
| | EX | Orange | 0.92 | 0.87 | 0.12 |
| | | Red | 0.76 | 0.91 | - |
| | MD | Orange | 0.80 | **0.58** | 0.20 |
| | | Red | **0.12** | 0.92 | - |
| | CI | Orange | 0.84 | 0.79 | - |
| | | Red | **0.52** | 0.94 | - |
| | EI | Orange | 0.76 | 0.75 | - |
| | | Red | **0.40** | 0.91 | - |
| **BAT12**[1] | Total | Orange | 0.88 | 0.78 | 0.08 |
| | | Red | **0.56** | 0.91 | - |
| **BAT4**[1] | | Orange | 0.96 | 0.72 | 0.26 |
| | | Red | **0.40** | 0.94 | - |

[1]BAT= Burnout Assessment Tool, long (BAT23) short (BAT12) and ultra-short (BAT4) version;

**Supplementary Table S2.** Percentages and counts of burnout complaints (and risk for clinical burnout) in a representative sample of Swedish work force (n=1603); green= no complaints (low risk of clinical burnout) orange= mild complaints (at risk for clinical burnout), red= severe complaints (very high risk for clinical burnout).

| BAT23[1] | | Swedish cut-off | | | Total |
|---|---|---|---|---|---|
| | | Green | Orange | Red | |
| | | % (n) | % (n) | % (n) | % (n) |
| Pooled cut-off[2] | Green | 73.4 (1177) | 6.7 (107) | 0.0 (0) | 80.1 (1284) |
| | Orange | 0.0 (0) | 6.6 (105) | 5.7 (92) | 12.3 (197) |
| | Red | 0.0 (0) | 0.0 (0) | 7.6 (122) | 7.6 (122) |
| Total | | 73.4 (1177) | 13.2 (212) | 13.3 (214) | 100.0 (1603) |

[1]BAT= Burnout Assessment Tool long (BAT23) [2]Pooled cut-off according to: Schaufeli, W. B., De Witte, H., Hakanen, J. J., Kaltiainen, J., & Kok, R. (2023). How to assess severe burnout? Cutoff points for the Burnout Assessment Tool (BAT) based on three European samples. Scandinavian Journal of Work, Environment and Health, 49(4), 293-302.

**Supplementary Table S3.** Percentages and counts of burnout complaints (and risk for clinical burnout) in a representative sample of Swedish work force (n=1603); green no complaints (low risk of clinical burnout) orange= mild complaints (at risk for clinical burnout), red= severe complaints (very high risk for clinical burnout).

| BAT12[1] | | Swedish cut-off | | | Total |
|---|---|---|---|---|---|
| | | Green | Orange | Red | |
| | | % (n) | % (n) | % (n) | % (n) |
| Pooled cut-off[2] | Green | 68.1 (1092) | 13.5 (217) | 0.0 (0) | 81.6 (1309) |
| | Orange | 0.0 (0) | 5.7 (92) | 5.5 (87) | 11.2 (179) |
| | Red | 0.0 (0) | 0.0 (0) | 7.2 (115) | 7.2 (115) |
| Total | | 68.1 (1099) | 19.2 (310) | 12.7 (205) | 100.0 (1603) |

[1]BAT= Short Burnout Assessment Tool long (BAT12), [2]Pooled cut-off according to: Schaufeli, W. B., De Witte, H., Hakanen, J. J., Kaltiainen, J., & Kok, R. (2023). How to assess severe burnout? Cutoff points for the Burnout Assessment Tool (BAT) based on three European samples. Scandinavian Journal of Work, Environment and Health, 49(4), 293-302.
